# Alternative Method for Glyphosate Determination in Unroasted Green Coffee Beans by Liquid Chromatography Tandem Mass Spectrometry (LC–MS/MS)

**DOI:** 10.1021/acs.jafc.4c06366

**Published:** 2024-10-24

**Authors:** Ana Carolina Pereira Paiva, Emanuel Carvalho de Assis, Leonardo d’Antonino, Maria Eliana Lopes Ribeiro de Queiroz, Antonio Alberto da Silva

**Affiliations:** † Department of Chemistry, Universidade Federal de Viçosa, Viçosa, Minas Gerais 36570900, Brasil; ‡ Department of Soil, Universidade Federal de Viçosa, Viçosa, Minas Gerais 36570900, Brasil; § Department of Agronomy, Universidade Federal de Viçosa, Viçosa, Minas Gerais 36570900, Brasil

**Keywords:** sample preparation, herbicide, Coffea arabica, method validation, residue monitoring, food safety

## Abstract

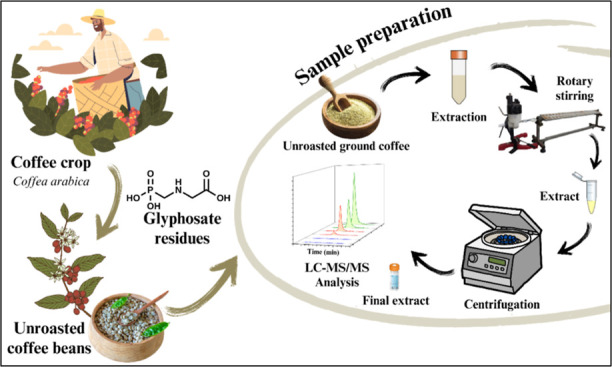

This research proposes an alternative method to detect and quantify glyphosate residues in unroasted green coffee beans by LC–MS/MS. The sample preparation was conducted without derivatization steps, with integrated cleanup, which improves the analytical method’s frequency. Validation results were consistent with the requirements of the regulatory guidelines employed. Specificity, linearity (*r*^2^ = 0.9991), precision (RSD ≤ 9%), and recovery (92–112%) were ensured, with a satisfactory limit of quantification (LOQ = 0.48 mg kg^–1^). These data demonstrate that the method is suitable for monitoring glyphosate residues in unroasted coffee beans while offering simplicity and speed in sample preparation. The method was applied to analyze authentic unroasted coffee bean samples, in which two of them were contaminated with glyphosate (<LOQ). These results exhibit the importance of glyphosate monitoring in coffee samples and enhance that the method can be successfully implemented as a tool to guarantee food quality and safety.

## 1 Introduction

Coffee is a globally consumed beverage. Brazil, the world’s largest coffee producer and the second-largest consumer, supplies approximately 38% of the international coffee market.^1^ The diversity of coffee characteristics and producers in Brazil results in high exportation levels of unroasted coffee directly to buyers, importers, and roasting companies. According to a report by the Brazilian Coffee Exporters Council (2024),^2^ more than 40,5 million bags of Arabica and Conillon coffee were exported in 2023.

To increase production and ensure coffee quality, pesticides are employed. These products function in pest and weed control and disease management, securing grain excellence while preserving the ease of the harvest process.^3^ Among the pesticides employed, glyphosate (*N*-(phosphonomethyl)glycine), a systemic, nonselective glycine derivative herbicide, is the most widely marketed active ingredient in the world for weed control.^4^ It is a polar molecule with low molecular mass, high water solubility, and negligible volatility (Table S1, Supporting Information). In coffee crops, the maximum residue limit (MRL) of glyphosate permitted is 1.0 mg kg^–1^ in Brazil and the United States and 0.1 mg kg^–1^ in the European Union.^5−7^

If not applied correctly and within the appropriate time frame, once on the surface of the leaves, glyphosate is absorbed by the plant and translocated via the phloem. Therefore, this herbicide is easily distributed throughout the plant due to the glyphosate’s hydrophilicity and ionization capacity.^8^ At the flowering and fruiting stages of the coffee crop, due to the significant movement of water and nutrients for fruit development and expansion, the translocation of glyphosate may lead to its accumulation in the coffee beans.^9,10^

Generally, glyphosate is indirectly analyzed in coffee crops due to the inherent complexity of both the matrix and the glyphosate analysis. The monitoring has been conducted through the plant’s physiological response,^11^ photosynthetic activity, and the amount of consumed carbon,^12^ as well as the quantity of shikimic acid.^13^

Although there is a lack of information concerning glyphosate monitoring in coffee crops, this herbicide is determined in other food samples using chromatographic techniques. Typically, a sample derivatization procedure is necessary, due to the polar and small nature of this molecule, making glyphosate suitable for fluorescence detection at liquid chromatography or even mass spectrometry for both gas and liquid chromatography.^14−16^ Nevertheless, derivatization extends the sample preparation time, requires adjustment of the reactivity medium’s pH, and may lead to the formation of byproducts that complicate the cleanup step and amplify equipment maintenance and cleanup procedures.^14,17,18^

New methods have been developed to analyze glyphosate in food matrices without the necessity of derivatization steps, reducing sample preparation time. The QuPPe method (quick polar pesticides method), developed by Anastassiades and colleagues (2019),^19^ aligns with this notion. This method extracts and analyzes highly polar pesticides in plant-based foods, including fruits, vegetables, cereals, legumes, grains, nuts, and, honey. It is a foundational method for glyphosate analysis in vegetables,^18,20^ fruits,^18,21,22^ oils,^23^ cereals such as wheat and oat,^22^ and honey.^24^ Martins-Júnior and colleagues (2009)^25^ proposed another glyphosate extraction method, an alternative to the QuPPe method, for efficient analysis of the herbicide residue by LC–MS/MS in soybean grains.

Although glyphosate determination is being conducted in various crops, to the best of our knowledge, there is no specific validated method for glyphosate analysis in unroasted coffee beans without derivatization steps. It is essential to monitor and determine the residue levels of glyphosate in this matrix to evaluate coffee quality and correlate glyphosate presence accurately. Moreover, glyphosate monitoring can allow more effective weed control in coffee production, ensuring zero or minimal residue levels.

Therefore, this study proposes an alternative, easily executable, simpler, efficient, precise, and faster method for quantifying glyphosate residues in unroasted green coffee beans using LC–MS/MS, without derivatization steps. The method was successfully applied to determine glyphosate residues in different samples collected from local producers of Minas Gerais, Brazil, enhancing the need to monitor this herbicide in coffee crops.

## 2 Material and Methods

### 2.1 Reagents and Solutions

Acetonitrile and methanol, both of LC–MS grade, were procured from Sigma-Aldrich. Formic acid (>98%, Sigma-Aldrich), dichloromethane (HPLC grade >99.8%, Sigma-Aldrich), ammonium formate (98%, Carlo Erba), and ammonium hydroxide (28–30%, Synth) were also employed in the experiments. Standard stock (500 and 100 mg L^–1^) and working solutions across different concentrations (500 μg L^–1^ to 10 mg L^–1^) of glyphosate (purity of 99% w/w—Sigma-Aldrich) and isotopically labeled glyphosate (^13^C, ^15^N) (purity of 99% w/w—Sigma-Aldrich) were prepared in ultrapure water and acetonitrile at a ratio of 90:10% (v/v). All reagents were used without any further purification steps.

### 2.2 Glyphosate Analysis by LC–MS/MS

For analysis, a liquid chromatograph coupled with a triple quadrupole mass spectrometer model LCMS 8040 Shimadzu, equipped with a SIL 10Ai autoinjector and an Electrospray Ionization (ESI) source was used. An analytical column Restek Raptor Polar X (30 mm L × 2.1 mm I.D. × 2.7 μm) maintained at 35 °C was employed, and the analyses were performed at a flow rate of 0.4 mL min^–1^. A sample injection volume of 5 μL was used. Two mobile phase compositions were evaluated. In the first binary composition, the mobile phase consisted of a 0.5% (v/v) aqueous solution of formic acid (phase A) and acetonitrile acidified with 0.5% (v/v) formic acid (phase B). The second binary composition consisted of a 0.63 g L^–1^ aqueous solution of ammonium formate and 0.05% (v/v) ammonium hydroxide (phase A) and 100% methanol (phase B).^25,26^ These phases were assessed in an isocratic mode (90% phase A and 10% phase B) to determine the composition that would promote optimal elution and ionization of the analyte at the ESI source, either in positive mode (M + H) or negative mode (M – H).

The elution of glyphosate was subsequently performed in a gradient analysis mode, with an initial composition of 40% A and 60% B held for 1.5 min, followed by a linear decay for 1 min to 5% B, maintained for 4.5 min, and then an immediate return to the initial composition (60% B) held for 3 min. The total run time was 10 min. The entire chromatographic system was passivated with Restek passivating solution [1760 μg mL^–1^ medronic acid in 50:50 methanol and water % (v/v)] before glyphosate analyses following the manufacturer’s recommendations. This process was carried out repeatedly as required. Nitrogen was used as the ESI source’s nebulization gas and drying gas, with flow rates maintained at 15.0 and 3.0 L min^–1^, respectively. The desolvation line and heating block temperatures were maintained at 280 and 450 °C, respectively. The mass spectrometer operated in multiple reaction monitoring (MRM) mode, with optimization of collision energy (CE) parameters, Q1 and Q3 quadrupole voltages, and adjustment of precursor and product ions for both glyphosate and isotopic labeled glyphosate (^13^C, ^15^N) (used as internal standard at validation procedure), using Shimadzu LabSolutions software (Table 1). Quantification was conducted using peak area, with three transitions for analyte identification and confirmation.

**Table 1:** Optimized Parameter Values for Glyphosate and Isotopically Labeled Glyphosate- ^13^C, ^15^N Analysis in MRM Mode (−) Performed Using LabSolutions Software (Shimadzu)

	precursor ion (*m*/*z*)	product ion (*m*/*z*)	CE^a^ (V)	Q1 (V)	Q3 (V)
glyphosate	168.1	63.1	26	10	10
	168.1	78.8	40	24	26
	168.1	149.9	14	17	28
glyphosate-^13^C, ^15^N	169.9	63.2	23	20	23
	169.9	78.9	37	28	13
	169.9	81.1	17	17	13

a Collision energy.

### 2.3 Glyphosate Determination in Unroasted Green Coffee Beans

For optimization and validation, this study utilized unroasted green coffee beans of the *Coffea arabica* species sourced from a local producer who does not employ herbicidal products containing glyphosate. The absence of the herbicide on the beans was confirmed by LC–MS/MS. These beans were depulped, dried, and unroasted. During the research, glyphosate solution was added at various concentrations to the samples.

The glyphosate extraction method proposed in this study was adapted from the method proposed by Martins-Júnior and colleagues (2009)^25^ for glyphosate determination in soybeans.

The optimized method for glyphosate extraction from coffee beans involved adding 2.0 g of crushed and sieved unroasted coffee bean samples to a 50 mL Falcon-type polypropylene tube, followed by adding 5.0 mL of dichloromethane and vortex agitation for 1 min. Subsequently, 10.0 mL of ultrapure water was added, followed by vortex agitation for 20 s, and then on a rotary shaker (360°) at 80 rpm for 30 min. Next, 400 μL of the supernatant was transferred to a new 1.5 mL Eppendorf-type tube, along with 600 μL of methanol, agitated by vortex for 10 s, and then centrifuged in a refrigerated centrifuge at 10 °C for 10 min at 15,000 rpm. The supernatant was filtered [polytetrafluoroethylene (PTFE) membrane; 0.45 μm] into a filtration vial and analyzed using LC–MS/MS.

### 2.4 Method Optimization

The effect of ultrapure water volume (10–30 mL), rotational agitation time on a rotary shaker (30–90 min), and rotation speed during agitation (40–80 rpm) on herbicide extraction was investigated using a 2^3^-factorial design with a central point and 3 repetitions. The influence of centrifugation speed (5000; 15,000 rpm) and centrifugation temperature (−10; 10 °C) at extract cleanup was examined using a 2^2^-factorial design without a central point, with 3 repetitions. In these experiments, crushed coffee bean samples were fortified with glyphosate at 1.0 mg kg^–1^ and immediately subjected to extraction. The extracts were filtered using a filtration vial (RC 0.45 μm) and analyzed by LC–MS/MS. Peak areas were used as the response in the factorial designs. All data were analyzed using Statistica 7 software at a 95% confidence level.

A univariate study evaluated the ratio of methanol volume to extract volume [50:50; 60:40; and 70:30% (v/v)] at two glyphosate concentration levels (0.5 and 1.0 mg kg^–1^) to obtain a more suitable final extract for chromatographic analysis. The chromatograms were compared, and area ratios of the glyphosate signal and the blank sample signal at the same retention time were employed as a response to define the optimal analytical condition.

### 2.5 Method Validation

Glyphosate quantification was carried out using the matrix overlay method with the addition of isotopically labeled glyphosate (^13^C, ^15^N) as an internal standard (IS). The method validation considered the parameters: specificity, linearity, and sensitivity, limit of detection (LOD), limit of quantification (LOQ), precision in terms of repeatability (intraday) and intermediate precision (interday), accuracy (recovery), matrix effect, robustness, extracted ion chromatogram (XIC), and ion intensity ratio. Procedures were conducted following the validation guidelines for pesticide residue analysis in food provided by SANTE/11312/2021^27^ and the Collegiate Board Resolution RDC no 166, provided by the Brazilian National Health Surveillance Agency (ANVISA),^28^ a regulatory body in Brazil.

Specificity was evaluated in terms of the response of glyphosate standard solution and matrix-fortified extract with glyphosate, based on the analyte’s retention time (RT), compared to the response of a blank matrix extract sample without the analyte at the same time.

Linearity and sensitivity were assessed by injecting triplicate extracts of fortified samples with glyphosate at 7 different levels (0.5–6.0 mg kg^–1^), including an internal standard (4.0 mg kg^–1^), to obtain the line equation and the coefficient of determination (*R*^2^). The linear range deviation was determined by the relative standard deviation of back-calculated concentrations from true concentrations.

The limits of detection (LOD) and quantification (LOQ) were defined as 3.3 and 10 times the ratio of the standard deviation of the blank (s) divided by the slope of the analytical curve (S).^28^

Precision was assessed for repeatability, conducted within a single day of analysis (intraday), and intermediate precision, with 3 nonconsecutive days of analysis (interday), using the relative standard deviation (RSD) (≤20%). These tests were performed by a single analyst at two distinct concentration levels [1.0 and 5.0 mg kg^–1^/maximum residue limit (MRL) and 5× MRL], with 5 replicates for each level. Accuracy was evaluated as the method’s recovery (% *R*) in tests conducted at two distinct levels (MRL and 5× MRL), with 5 replicates for each level. The concentration levels were defined based on the MRL for both accuracy and precision since the MRL is the target concentration for determining sample quality. The calculated concentration averages were used as the response for these essays.

The matrix effect was assessed by comparing an analytical curve prepared in ultrapure water and 10% acetonitrile with an analytical curve prepared in matrix extract (5–120 μg L^–1^; internal standard at 80 μg L^–1^). The percentage matrix effect (% ME) was determined using the slope of the obtained analytical curves, employing eq 1
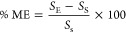
1where *S*_E_ represents the slope of the analytical curve prepared in the matrix extract, and *S*_S_ is the slope of the analytical curve prepared in solvents.

Robustness was assessed in terms of the rotational agitation time on a rotary shaker (28 and 32 min), centrifugation time in a refrigerated centrifuge (8 and 12 min), and chromatographic column temperature (33 and 37 °C). Glyphosate-free samples were fortified with 1.0 mg kg^–1^ (MRL) and subjected to the optimized extraction process. The calculated glyphosate concentrations were taken as the response, and the effects of each parameter were determined using eq 2
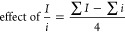
2where ∑*I* represents the sum of glyphosate concentrations obtained in experiments conducted with higher values of the selected parameters, and ∑*i* is the sum of glyphosate concentrations obtained in experiments conducted with lower values of the selected parameters.

The standard deviation of differences (SD_Di_) for the effects of the evaluated parameters was calculated (eq 3) using the Youden approach,^29^ for comparison with the repeatability standard deviation.
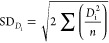
3where *D*_i_^2^ represents the squared differences of the selected parameters, and *n* is the number of parameters.

The full overlap of the glyphosate and glyphosate-^13^C, ^15^N peaks were verified for each selected transition by comparing the extracted ion chromatograms (XIC). The relative ion ratio intensities (fragments) selected in the standard solution were compared to the responses obtained at analyte samples from the matrix extract. The validation guideline requires a deviation within ±30% between standard solution and matrix extract ion ratio intensities.^27^

## 3 Results and Discussion

### 3.1 Glyphosate Analysis by LC–MS/MS

**Figure 1:**
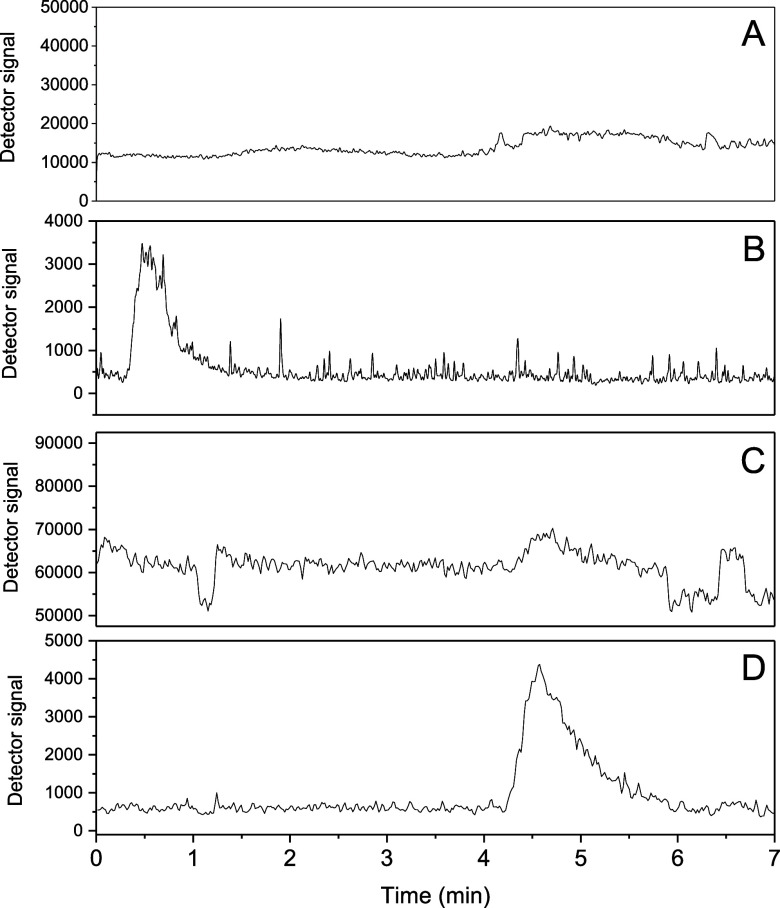
Chromatograms obtained after the analysis of a 500 μg L^–1^ glyphosate standard solution using isocratic elution (flow rate 0.4 mL min^–1^) with the following mobile phase compositions and analysis modes: (A) 90% aqueous solution of 0.63 g L^–1^ ammonium formate and ammonium hydroxide 0.05%(v/v) and 10% methanol (SIM + Mode); (B) 90% aqueous solution of 0.63 g L^–1^ ammonium formate and ammonium hydroxide 0.05% (v/v) and 10% methanol (SIM – mode); (C) 90% aqueous solution of formic acid 0.5% (v/v) and 10% acetonitrile acidified with formic acid 0.5% (v/v) (SIM + mode); (D) 90% aqueous solution of formic acid 0.5%(v/v) and 10% acetonitrile acidified with formic acid 0.5% (v/v) (SIM – mode).

Two mobile phase compositions were evaluated, with injections in SIM mode (M + H)^+^ and (M – H)^−^ at isocratic mode, to determine the optimal ionization condition for glyphosate, as this molecule can be positively and negatively ionized in the ESI source. Figure 1 presents the chromatographic response for the evaluated compositions. It was observed that the solution of formic acid 0.5% (v/v) (phase A) and acetonitrile acidified with formic acid 0.5% (v/v) (phase B), along with the negative ionization mode (chromatogram D), yielded the best response, with a glyphosate’s retention time of 4.6 min, approximately. In this composition, the pH of the mobile phase is approximately 2.3.

The glyphosate molecule exhibits acid–base behavior in aqueous media. To justify the enhanced ionization in the negative mode, one can assess the value of the effective charge (*q*_ef_) of this species at this pH, which is determined by the sum of the species’ charge multiplied by its equilibrium fraction (eq 4).^30^
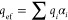
4Where *q*_*i*_ represents the charge of the chemical species, and α_*i*_ is the equilibrium fraction of the species at a specific pH value.

Glyphosate effective charge of −0.29 at pH 2.3 (Figure S1, Supporting Information)^31^ indicates that predominantly, the charge of acid–base species present in the medium will be negative at this pH value, favoring ionization through proton loss in the ESI source.

**Figure 2:**
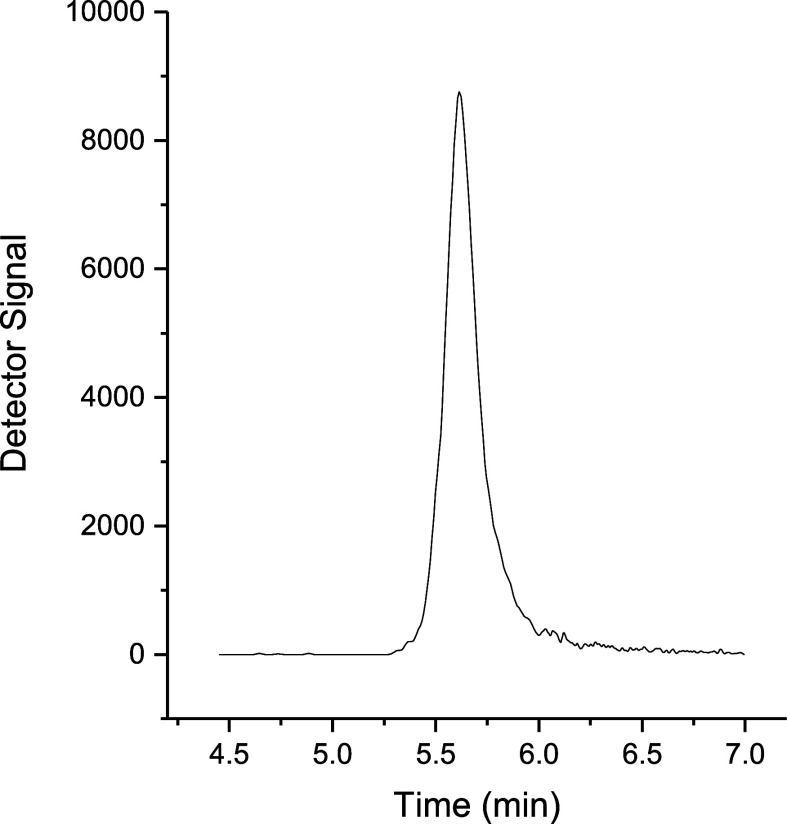
Chromatogram obtained after analysis of a 500 μg L^–1^ glyphosate standard solution at gradient mode.

The MRM mode was optimized using this mobile phase composition. Transitions 168.1 > 63.1, 168.1 > 78.8, and 168.1 > 149.9 *m*/*z* were selected for glyphosate identification and quantification, ensuring higher sensitivity and specificity of the analyses. The optimal peak shape was achieved with gradient elution (Figure 2). Glyphosate’s retention time was adjusted to 5.6 min in this mode. As the Restek Raptor polar X column utilizes a stationary phase composed of porous silica particles bonded with a polar X ligand (not disclosed by the manufacturer), it can interact hydrophilically with the analyte (HILIC chromatography) and perform ion exchange based on the polarity of the mobile phase during the chromatographic run. Hence, it is necessary to initially have a higher percentage of organic solvent in the mobile phase, ensuring greater interaction of the analyte with the stationary phase (HILIC retention), and throughout the chromatographic run, there is an increase in the aqueous fraction, allowing for the elution of the herbicide (ion exchange mechanism).^32^

### 3.2 Optimization of the Glyphosate Determination Method in Unroasted Green Coffee Beans

The effects of the parameters such as volume of ultrapure water as the extracting solvent, time, and rotational speed of the rotary shaker were evaluated. The areas obtained from the chromatographic analyses were used as the response to the employed factorial design. The results obtained indicated that only the volume of water was significant (positively) in the extraction of glyphosate (Figure S2, Supporting Information).

A smaller volume of extraction solvent (10.0 mL) was able to promote herbicide extraction with less analyte dilution, resulting in a larger chromatographic peak area. A new analysis of the data was performed using stepwise regression, which systematically adds and removes variables of higher and lower significance, respectively. This further analysis of the results aimed to determine the best fit of the employed model and assess the effect of the rotation speed factor and its interaction with the water volume factor. After successive removal of less significant factors, it was observed that rotation speed is a nonsignificant factor for the extraction technique (p-value = 0.06). However, a higher rotation speed (80 rpm) was selected for future experiments, given the average signal gain of 9% observed under this condition when compared to rotation at 40 and 60 rpm.

The effects of the parameters rotation speed and centrifugation temperature were analyzed in a new factorial design. Rotation speed during the centrifugation step was found to be significantly positive for herbicide extraction (*p* < 0.05) (Figure S3, Supporting Information).

By increasing the rotation speed during centrifugation to 15,000 rpm and cooling the sample at 10 °C, the solubility of particles in the matrix extract is reduced, making it easier to precipitate them.^19^ Thus, the matrix effect is diminished, ensuring a cleaner extract, which contributes to extending the chromatographic column’s lifespan.

Due to the complexity of the coffee matrix, dichloromethane was employed to reduce the coextraction of some compounds, such as lipids, and purify the extract.^25^ Methanol was employed with de same intent. In this case, methanol contributed to protein precipitation in the final sample extract.^25^ To better preserve the chromatographic system, and to avoid the increase of the system pressure after sequential extract analysis, final diluted with methanol extracts were evaluated.

Regarding this alteration of the ratio between the volume of methanol and the volume of extract used, the analysis of chromatograms revealed the presence of the glyphosate peak in samples fortified at 1.0 mg kg^–1^ in all three evaluated proportions. However, in the extracts corresponding to samples fortified at 0.5 mg kg^–1^, the peak was observed in the 50:50 and 60:40% (v/v) proportions, with a glyphosate signal 23 and 10 times higher, respectively, than the blank extract signal. In contrast, the glyphosate signal was very similar to the baseline noise at the 70:30% (v/v) proportion of methanol and extract, with a signal ratio between the sample and the blank extract lower than 5. Consequently, the choice was made to use the 60:40% (v/v) proportion to obtain a final extract that is better suited for preserving the chromatographic system. At this condition, as the glyphosate signal is at least 10 times higher than the blank extract signal, is possible to guarantee glyphosate identification and possible quantification at concentrations of interest and lower than the MRL.

### 3.3 Validation of Glyphosate Determination Method in Unroasted Green Coffee Beans

The quantification of glyphosate was performed using the matrix-matched calibration method. The procedure for the calibration curve construction and validation of the method involved fortifying samples of glyphosate-free green coffee beans with various amounts of glyphosate and a fixed concentration of glyphosate- ^13^C, ^15^N (IS). Subsequently, these samples were subjected to the optimized extraction method, and the resulting extracts were filtered and analyzed by LC–MS/MS. This approach compensates for the potential matrix interferences in glyphosate quantification.^27^

#### 3.3.1 Specificity

**Figure 3:**
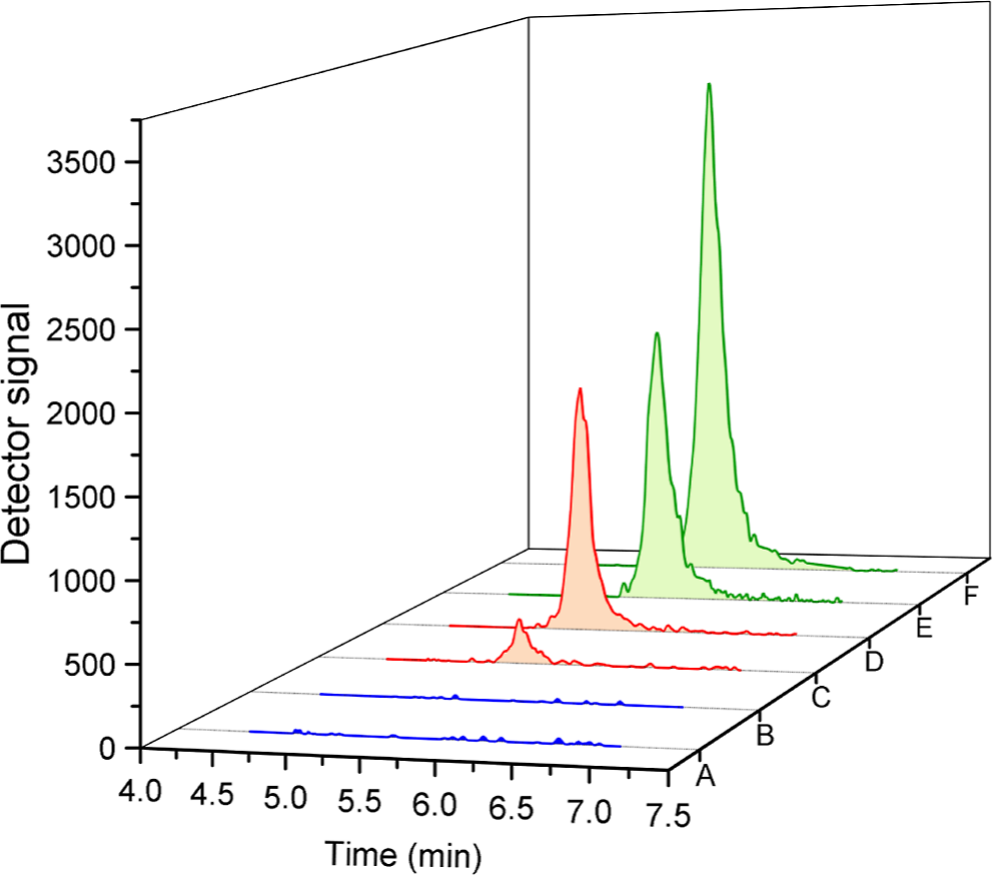
Chromatograms obtained after analysis of glyphosate standard solution (E) and glyphosate- ^13^C, ^15^N (F), both at 100 μg L^–1^ (in green); matrix extract fortified with glyphosate (C) at 1.0 mg kg^–1^ and with glyphosate- ^13^C, ^15^N (D) at 4.0 mg kg ^–1^ (in red) and matrix extract without the presence of the herbicide (A and B, in blue).

The method’s specificity is assessed to ensure that the detector provides a signal that effectively and exclusively identifies the analyte. The evaluation of specificity was conducted by comparing the chromatograms obtained from the analysis of blank and fortified matrix extracts with glyphosate, as well as the standard solution of the herbicide (Figure 3).

No peak was observed in the analysis of the glyphosate-free extract at the same retention time as the analyte [RT (gly) = 5.4 min) and the IS used (RT (gly-^13^C ^15^N) = 5.5 min], ensuring the specificity of the method. The noise area in the blank (*A* = 32) is equivalent to 0.19% of the analyte area (*A* = 17,058), and this result is by the requirement of the validation guideline (≤30%).^27^

#### 3.3.2 Linearity, Sensitivity, Limit of Detection, and Limit of Quantification

Linearity and sensitivity were assessed by analyzing the analytical curve. The linear equation obtained was *A*/*A*_IS_ = 0.1520 C (gly) + 0.0011, with a coefficient of determination (*R*^2^) of 0.9991. This value surpasses the minimum requirement of 0.990 set by ANVISA.^28^

The linear range deviation determined by the relative standard deviation of calculated concentration from true concentration was determined by the analytical curve, which should be ≤ ± 20%.^27^ Values ranging from 0.49% to 8.62% were obtained (Table S2, Supporting Information), following the validation guideline requirements.

**Figure 4:**
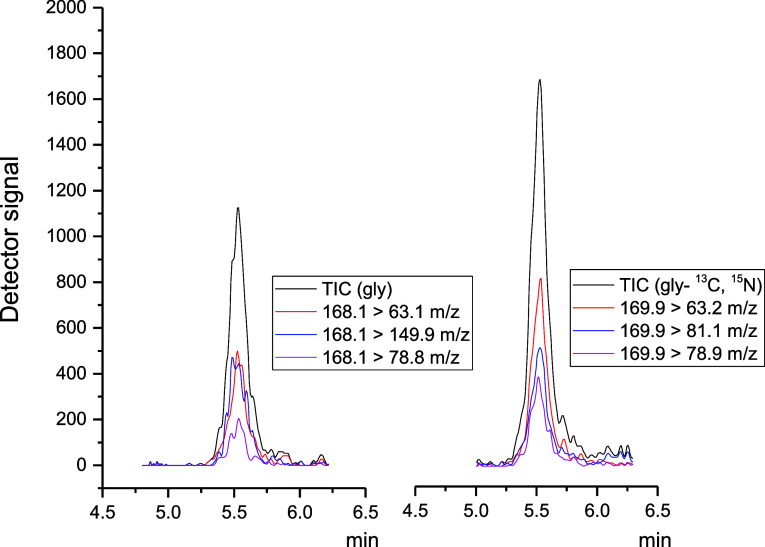
Total ion chromatogram (TIC) and extracted ion chromatograms (XIC) obtained after the analysis of the matrix extract fortified at 4.0 mg kg^–1^ with glyphosate and glyphosate- ^13^C, ^15^N solution. On the left, chromatograms for glyphosate and its selected transitions 168.10 > 63.10; 168.10 > 149.90; 168.10 > 78.80 *m*/*z* are presented. On the right, chromatograms for glyphosate- ^13^C, ^15^N and its selected transitions 169.90 > 63.20; 169.90 > 81.10; 169.90 > 78.90 *m*/*z* are presented.

In this study, the limits of detection and quantification were found to be 0.16 and 0.48 mg kg^–1^, respectively. The sensitivity of the mass spectrometer was assessed by checking the full overlap of the extracted ion chromatograms (XIC) of glyphosate and glyphosate- ^13^C, ^15^N (Figure 4), to ensure proper identification of these compounds. The deviation between the standard solution and matrix extract ion ratio intensities was up to −25.9%, following validation guidelines.

#### 3.3.3 Repeatability, Intermediate Precision, and Recovery

The precision considering repeatability and intermediate precision were 5.25–8.54% and 5.74–9.05%, respectively, expressed in terms of relative standard deviation (RSD). It is worth noting that the values obtained were below the recommended 20% as stated in the SANTE/11312/2021^27^ (Table 2). The recovery varied from 92.08 to 112.01%. According to the same guideline, recovery should fall between 70 and 120%, a range that encompasses the results obtained in this study. Recovery was also monitored throughout the entire validation process to ensure that the method consistently provides acceptable recovery values. Across the conducted studies, % *R* values remained between 80.09 and 112.01%, meeting the guideline’s requirements for method validation.

**Table 2:** Parameters for Method Validation: Linear Regression, Coefficient of Determination (*R*^2^), Linear Range, Linear Range RSD (%), Limit of Quantification (LOQ), Fortification Level (FL), Intra-day and Inter-day Precision in Terms of Relative Standard Deviation (RSD_r_), and Percentage of Recovery and Standard Deviation (% *R* ± SD) of Glyphosate in Green Coffee Beans Samples

linear regression/*R*^2^	linear range (mg kg^–1^)	linear range RSD (%)	LOQ (mg kg^–1^)	FL	intraday RSD_r_ (%) (*n* = 5)	interday RSD_r_ (%) (*n* = 15)	% *R* ± SD
*y* = 0.1520*x* + 0.0011	0.50–6.00	0.49–8.62%	0.48	1.00	8.45	9.05	112.01 ± 0.01
0.9991				5.00	5.25	5.74	92.08 ± 0.04

#### 3.3.4 Matrix Effect

The matrix effect ME is expressed as a percentage (% ME) and measures how the presence of sample coextracts affects the analyte response. This effect can either increase or decrease the analyte response in the matrix extract compared to its response in a pure solvent solution.^27^ The calibration curve equation for pure solvent was determined as *A*/*A*_IS_ = 0.0068 C(gly) + 0.0057, with an *R*^2^ value of 0.9919. For the calibration curve in the matrix extract, the equation was *A*/*A*_IS_ = 0.0064 C(gly) + 0.0113, with an *R*^2^ value of 0.9965 (Figure S4, Supporting Information).

Both curves exhibited similar angular coefficients, with *S*_(Matrix)_ = 0.0064 and *S*_(Solvent)_ = 0.0068. The percentage matrix effect was calculated, resulting in a % ME = −6.53%, indicating a decrease in glyphosate response when present in the extract. However, this effect is minimal and nonsignificant, as it is less than ±10%.

#### 3.3.5 Robustness

In the robustness analysis, the effects of agitation time (−0.0364), centrifugation time (−0.0024), and column temperature (−0.0251) parameters on the chromatographic response were evaluated.

For a more comprehensive evaluation of the results, the standard deviation of the differences was calculated according to the Youden approach (eq 3). A standard deviation of SDD__i__ = 0.0361, was obtained, which is lower than the standard deviation values obtained in the repeatability test SD_R_ = 0.0947. This result indicates that the assessed parameters did not influence the glyphosate response, confirming the robustness of the method used.

All the validation parameters assessed yielded results that met the requirements of both employed guidelines. It is important to emphasize that, to the best of our knowledge, there is no specific method in the literature for detecting and quantifying glyphosate residues in green coffee beans without derivatization steps. Therefore, the development of an appropriate and effective method for glyphosate analysis in green coffee beans is crucial to enable the monitoring of the herbicide residues in this matrix.

### 3.4 Comparison with the Literature

Glyphosate determination in unroasted green coffee beans was evaluated with the herbicide derivatization. A methodology was proposed by Jian-Lin and colleagues (2020)^33^ for this purpose, involving sample derivatization with fluorenylmethoxycarbonyl chloride (FMOC-Cl) and chromatographic analysis (UPLC-MS/MS). The authors obtained a limit of quantification of 0.05 mg kg^–1^ and reported satisfactory results for precision (RSD < 4.25%) and recovery (99.6–107.6%). However, this method presents a higher cost and lower analytical frequency, given that the derivatization agent FMOC involves a considerable cost (U$84/g) and total derivatization reaction time reached 6 h. In this context, methods that do not require derivatization are advantageous, as they allow lower analysis costs and require less time for sample preparation, leading to increased analytical frequency, as demonstrated by the method proposed in this study.

In a study conducted by Schrübbers and colleagues (2016),^36^ glyphosate analysis was performed directly on coffee leaves treated with different doses of the herbicide and leaves from areas (field) with different histories of glyphosate use. Sample preparation involved derivatization with FMOC, and the extracts were analyzed by LCMS. The authors obtained a limit of quantification of 0.30 mg kg^–1^, recovery rates between 101 and 107%, and precision <15% in terms of relative standard deviation (RSD). The analysis of treated leaves indicated an increase in glyphosate concentration with the increase in the applied dose, while the leaves collected from the field presented low glyphosate concentration (<LOQ). These results emphasize the absorption and translocation of glyphosate within coffee plants. Nevertheless, they did not ascertain whether glyphosate accumulates in coffee beans, as they did not assess the presence or absence of the herbicide within this matrix.

Given the scarcity of studies on glyphosate analysis in coffee beans, a comparison can be made among similar methodologies. Therefore, the study conducted by Martins-Junior and colleagues (2009)^25^ stands out, aimed at determining glyphosate in soybeans, where researchers employed a sample preparation similar to that presented in this study. Although the grains differ primarily in the number of proteins (coffee: 8.5–12.5% of proteins; soybeans: 38.9–41.8% of proteins),^34,35^ both validated methods present similar limits of quantification (coffee: LOQ = 0.48 mg kg^–1^; soybeans: LOQ = 0.30 mg kg^–1^), with good recovery and precision (coffee: 92.1–112.0% of recovery, RSD ≤ 9.05%; soybeans: 79.6–109.0% of recovery; RSD ≤ 12.2%).

### 3.5 AMPA Determination in Coffee Matrix

Plant metabolism can generate various metabolic products from herbicides. In this context, glyphosate’s primary metabolite, aminomethyl phosphonic acid (AMPA), is frequently analyzed alongside glyphosate.

Nonetheless, in fortified coffee extracts prepared and analyzed under the previously described chromatographic conditions, no AMPA peak was observed. A similar observation was reported by Cutillas and Fernández-Alba (2021),^37^ in their evaluation of glyphosate and AMPA residues in fruits and vegetables. The researchers used the same chromatographic column as presented in this study, the Restek Raptor Polar X. This column is available in three different column sizes: 2.7 μm of particle size (PZ), 30 mm length (L), and 2.1 mm of internal diameter (ID); 2.7 μm PZ, 50 mm L, and 2.1 mm ID; and 2.7 μm PZ, 50 mm L, and 3.0 mm ID. The authors found that when using the 30 mm length column, AMPA did not eluate in orange and quince matrices. Due to the limitation of the column available, comprehensive AMPA monitoring could not be conducted throughout this study.

### 3.6 Application of the Method in the Analysis of Authentic Unroasted Green Coffee Bean Samples

After method optimization and validation, it was applied to the analysis of authentic green coffee bean samples. Six distinct samples were acquired from coffee farmers in the Viçosa Microregion, located in the Zona da Mata region of Minas Gerais, Brazil.

The samples were prepared in triplicate following the optimized extraction method. The extracts were analyzed using LC–MS/MS, and the obtained responses were evaluated using a matrix-matched calibration on the same day. Among the collected samples, residues of glyphosate were detected in 2 samples, below the limit of quantification (<LOQ). These results highlight the importance of glyphosate residue monitoring in unroasted coffee beans. The characteristics of the proposed method, such as great validation results, no derivatization steps, and a reduced sample preparation time, make the method suitable for screening and routine analysis.

## Data Availability

Data will be made available on request.

## Supporting Information Available

Figure S1—Effective charge value for glyphosate molecule × pH. Figure S2—Pareto chart representing the effect of the factors water volume (mL), agitation time (min), and rotation speed (rpm) on glyphosate extraction from coffee beans. Figure S3—Pareto chart representing the effect of the factors rotation speed (rpm) and centrifugation temperature (°C) on glyphosate extraction from coffee beans. Figure S4—Glyphosate analytical curves prepared by fortifying the matrix extract (in red) and in a mixture of water and acetonitrile 90:10% (v/v) (in black) at different concentrations. Table S1—Some chemical and physical properties of glyphosate molecule. Table S2—True and back-calculated glyphosate concentration in green coffee bean samples obtained through the linear equation. The standard deviation and relative standard deviation are respectively shown for each concentration or point of the calibration curve (PDF)
