# Supplementary material for: Alternative Method for Glyphosate Determination in Unroasted Green Coffee Beans by Liquid Chromatography Tandem Mass Spectrometry (LC–MS/MS)
Source: J Agric Food Chem. 2024 Oct 24;72(47):26098–105. doi: 10.1021/acs.jafc.4c06366 (PMC11613452; doi:10.1021/acs.jafc.4c06366)
Supplement: Supplementary file 1 — jf4c06366_si_001.pdf [file jf4c06366_si_001.pdf]

AN ALTERNATIVE METHOD FOR GLYPHOSATE DETERMINATION IN UNROASTED GREEN COFFEE BEANS BY LIQUID CHROMATOGRAPHY TANDEM MASS SPECTROMETRY (LC-MS/MS)

Ana Carolina Pereira Paiva[a]; Emanuel Carvalho de Assis[a]; Leonardo d'Antonino[b]; Maria Eliana Lopes Ribeiro de Queiroz*[a]; Antonio Alberto da Silva[c]

[a] Department of Chemistry, Universidade Federal de Viçosa, Viçosa, Minas Gerais 36570900, Brasil

[b] Department of Soil, Universidade Federal de Viçosa, Viçosa, Minas Gerais 36570900, Brasil

[c] Department of Agronomy, Universidade Federal de Viçosa, Viçosa, Minas Gerais 36570900, Brasil

* Corresponding author

Maria Eliana Lopes Ribeiro de Queiroz

Department of Chemistry, Universidade Federal de Viçosa, Viçosa, Minas Gerais, 36570900, Brasil

E-mail: meliana@ufv.br
Phone: +55 (31) 3612 6631

Supporting Information

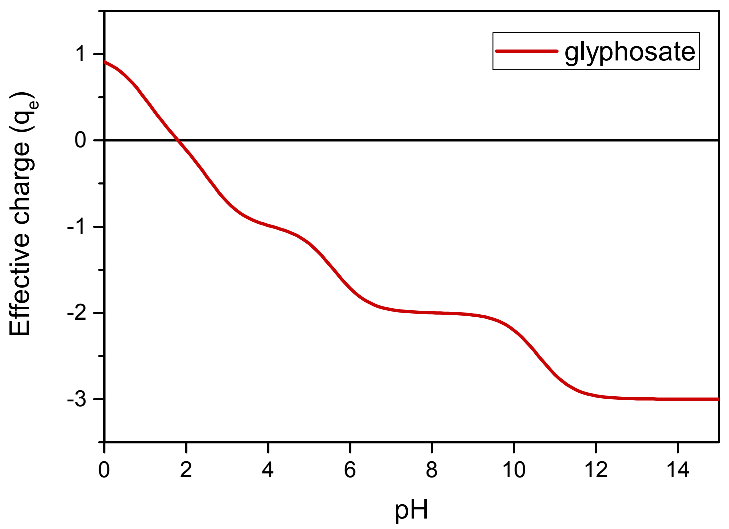

Figure S1 – Effective charge value for glyphosate molecule x pH.

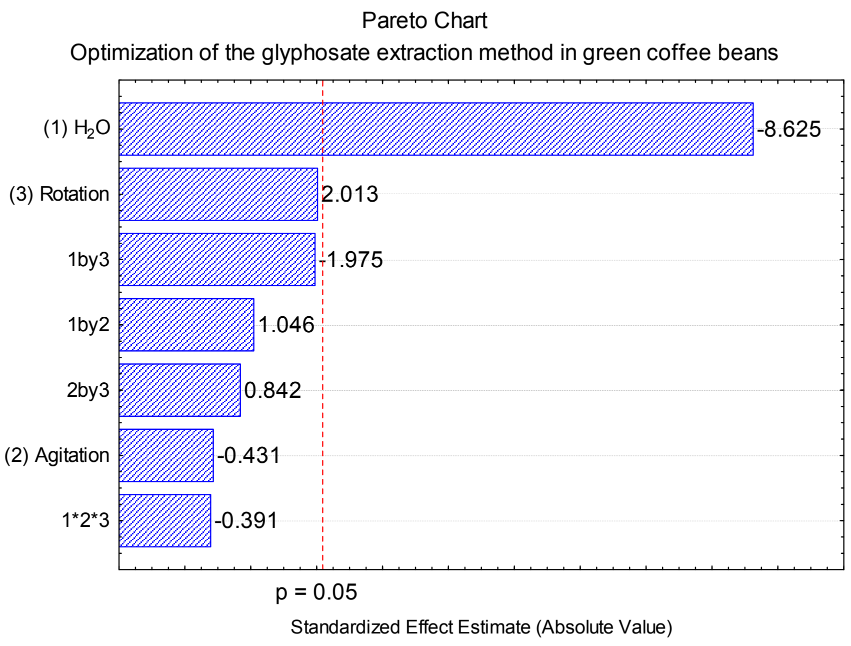

Caption:

H_2O Volume (mL): 10,0 (-); 20,0 (0); 30,0 (+)

Agitation time (min): 30 (-); 60 (0); 90 (+)

Rotation Speed (rpm): 40 (-); 60 (0); 80 (+)

Figure S2 - Pareto chart representing the effect of the factors water volume (mL), agitation time (min), and rotation speed (rpm) on glyphosate extraction from coffee beans.

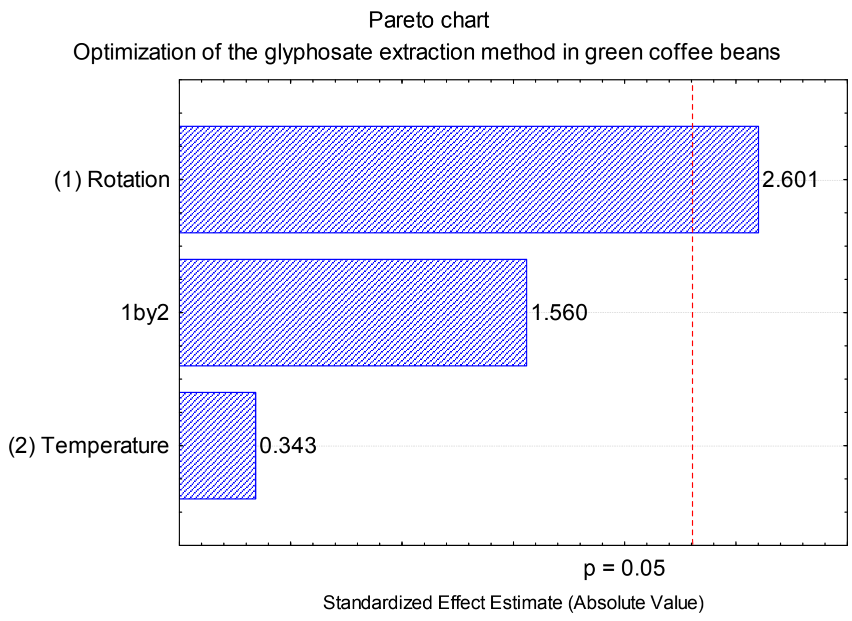

Caption:

Rotation speed (rpm): 5000 (-); 15000 (+)

Centrifugation temperature (°C): -10 (-); 10 (+)

Figure S3 – Pareto chart representing the effect of the factors rotation speed (rpm) and centrifugation temperature (°C) on glyphosate extraction from coffee beans.

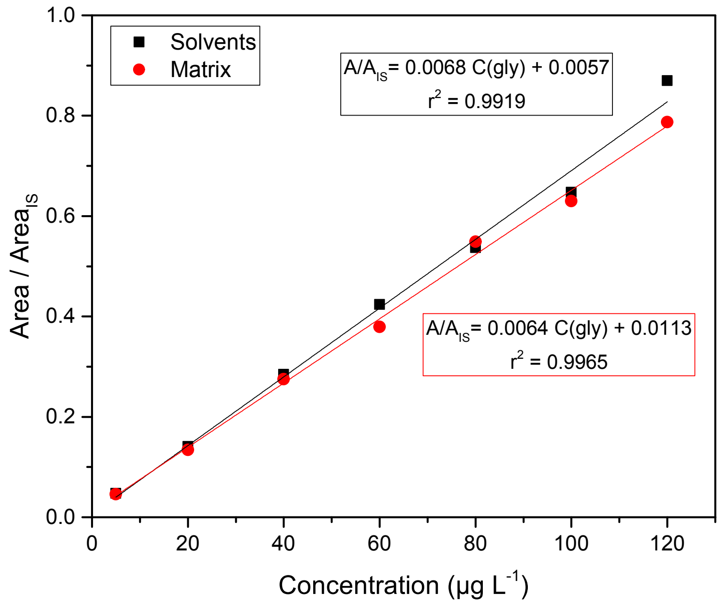

Figure S4 - Glyphosate analytical curves prepared by fortifying the matrix extract (in red) and in a mixture of water and acetonitrile 90:10 %(v/v) (in black) at different concentrations.

Glyphosate Physical-Chemical Properties	
Structure	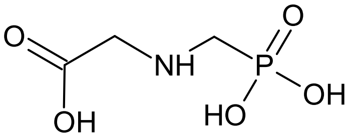
Molecular Weight	169.07 g mol^{-1}
Solubility in water (20 ºC)	1050 g L^{-1}
Vapor Pressure (20 ºC)	negligible
Density	1.7 g cm^{-3}
Dissociation constant (pKa)	$2.0_{(1)}$; $2.6_{(2)}$; $5.6_{(3)}$; $10.6_{(4)}$
Volatility	negligible

Source: PubChem, 2022; Paiva, 2023 (ACD/ChemSketch 2019.1.2).

Table S1 – Some chemical and physical properties of glyphosate molecule.

Actual concentration (mg kg^{-1})	Average calculated concentration (mg kg^{-1})	Standard deviation	Coefficient of variation (%)
0.50	0.48	0.04	8.62
1.00	0.98	0.01	0.49
2.00	1.98	0.02	1.17
3.00	3.04	0.17	5.62
4.00	4.11	0.10	2.38
5.00	4.92	0.24	4.86
6.00	5.98	0.25	4.18

Table S2 - True and back-calculated glyphosate concentration in green coffee bean samples obtained through the linear equation. The standard deviation and relative standard deviation are respectively shown for each concentration or point of the calibration curve.
